# Pore types, genesis, and evolution model of lacustrine oil-prone shale: a case study of the Cretaceous Qingshankou Formation, Songliao Basin, NE China

**DOI:** 10.1038/s41598-022-21154-y

**Published:** 2022-10-14

**Authors:** Wenyuan He, Bo Liu, Mengdi Sun, Liu Wang, Jinyou Zhang, Qamar Yasin, Shansi Tian, Shuo Gao, Chima Finnian Ukaomah

**Affiliations:** 1 Heilongjiang Provincial Key Laboratory of Continental Shale Oil, Daqing, 163712 Heilongjiang China; 2 Daqing Oilfield Limited Company, Daqing, 163002 Heilongjiang China; 3 Key Laboratory of Continental Shale Hydrocarbon Accumulation and Efficient Development, Ministry of Education, Northeast Petroleum University, Daqing, 163318 Heilongjiang China; 4 Exploration and Development Research Institute of Daqing Oilfield Co Ltd., Daqing, 163712 Heilongjiang China

**Keywords:** Solid Earth sciences, Geology, Mineralogy, Sedimentology

## Abstract

A comprehensive characterisation of the pore structure in shale oil reservoirs is essential for forecasting oil production and exploration risks. This study forecasted these risks in the oil-rich Songliao Basin using combination of high-resolution field emission scanning electron microscopy and quantitative X-ray diffraction to analyze the pore genesis and evolution mode within the first member of the Cretaceous Qingshankou Formation (K_2_qn_1_). The results showed the dominance of inorganic pores over organic pores, wherein diagenetic processes, such as compaction, pressure solution, and cementation, were responsible for the destruction of pore structure in the formation. Notably, the pores formed by dissolution and shrinkage cracks resulting from clay mineral transformation improved the oil storage space. Furthermore, according to the geochemical data and clay composition, the K_2_qn_1_ shale is in the middle diagenetic stage A, which can be further subdivided into A1 and A2 stages from top to bottom. The porosity slowly decreased in both sub-stages A1 and A2, wherein the decrease was stable in the latter. The diagenetic observations in this study are significant for the exploration of unconventional shale oil in petroliferous basins worldwide.

## Introduction

Shale oil is a source reservoir hydrocarbon accumulation that generates from and accumulates in oil-prone organic-rich shale, and huge oil reserves have been discovered in the marine and terrestrial shales of North America and China^1,2^. The development of shale oil exploration is driven by the increasing demand for global energy and decreasing production off conventional oil and resources^1^. This is exemplified by the fact that 70% of the proven conventional oil reserves in the petroliferous Songliao Basin of China, requires the exploration of unconventional shale oil to increase the 6.8 billion tons of proven oil reserves in the basin^3,4^. Although several studies have suggested the large potential reserves of shale oil present in the basin^5,6^, relatively little exploration has occurred.

Lacustrine shale oil reserves in China are estimated to be 130 billion tons^1^, and studies on lacustrine shale oil reservoirs in China reveal a dominance of semi-deep to deep lake facies contrasting from the dominance of shelf facies in the Bakken and Eagle Ford shale reservoirs of North America. The reservoirs in both China and North America are characterised by a matrix and microfracture reservoir space^7^. Previous studies on shale oil occurrence in the Songliao Basin have revealed the presence of shale oil reserves in the organic-rich lacustrine shales of Nenjiang and Qingshankou formations within the basin^1,7,8^. However, studies have revealed that the majority of shale oil flows produced from the basin are derived from wells drilled into the Qingshankou Formation at the Changling and Gulong depressions^1^. Although studies have experimentally characterized the excellent oil preservation conditions in the shales of Qingshankou Formation of the Changling sag^3,4^, the conditions in the Gulong depression remain unexplored.

The Gulong sag of the Daqing oil field comprises thick organic-rich shale deposits with moderate maturity representing an economically significant shale oil reservoir in the basin^8–10^. These shales have traditionally been considered as source rocks and have not been studied from the perspective of oil and gas reservoirs, and systematic research has not been conducted on pore types and the evolution of pore systems^11,12^. The major factors controlling the development of pores in these lacustrine shales and their influence on the shale oil development remain unclear. The aforementioned problems restrict the optimization of favourable areas and targets for shale oil in the Gulong sag^11,13^.

Recent, studies have revealed that one method for resolving the problems encountered during oil production from Gulong shale oil reservoir is the characterisation of organic matter and mineral genesis in the shale reservoir, which aids in understanding the factors that influence the oil abundance, reservoir availability, sensitivity, and compressibility^5^. Thus, studying the paleo-environment and the mechanism of organic enrichment in lacustrine fine-grained rocks can provide a thorough understanding of the characteristics and distribution of high-quality shale reservoirs^14^.

This study aims to resolve shale oil production problems in the Gulong shale oil reservoir through the experimental analysis of oil-prone shale samples via X-ray diffraction (XRD) and field emission scanning electron microscopy (FE-SEM). The organic matter enrichment and mineral and pore evolution mechanisms obtained in this study will provide valuable insights about oil production from lacustrine shale oil reservoirs worldwide.

## Geologic setting

**Figure 1:**
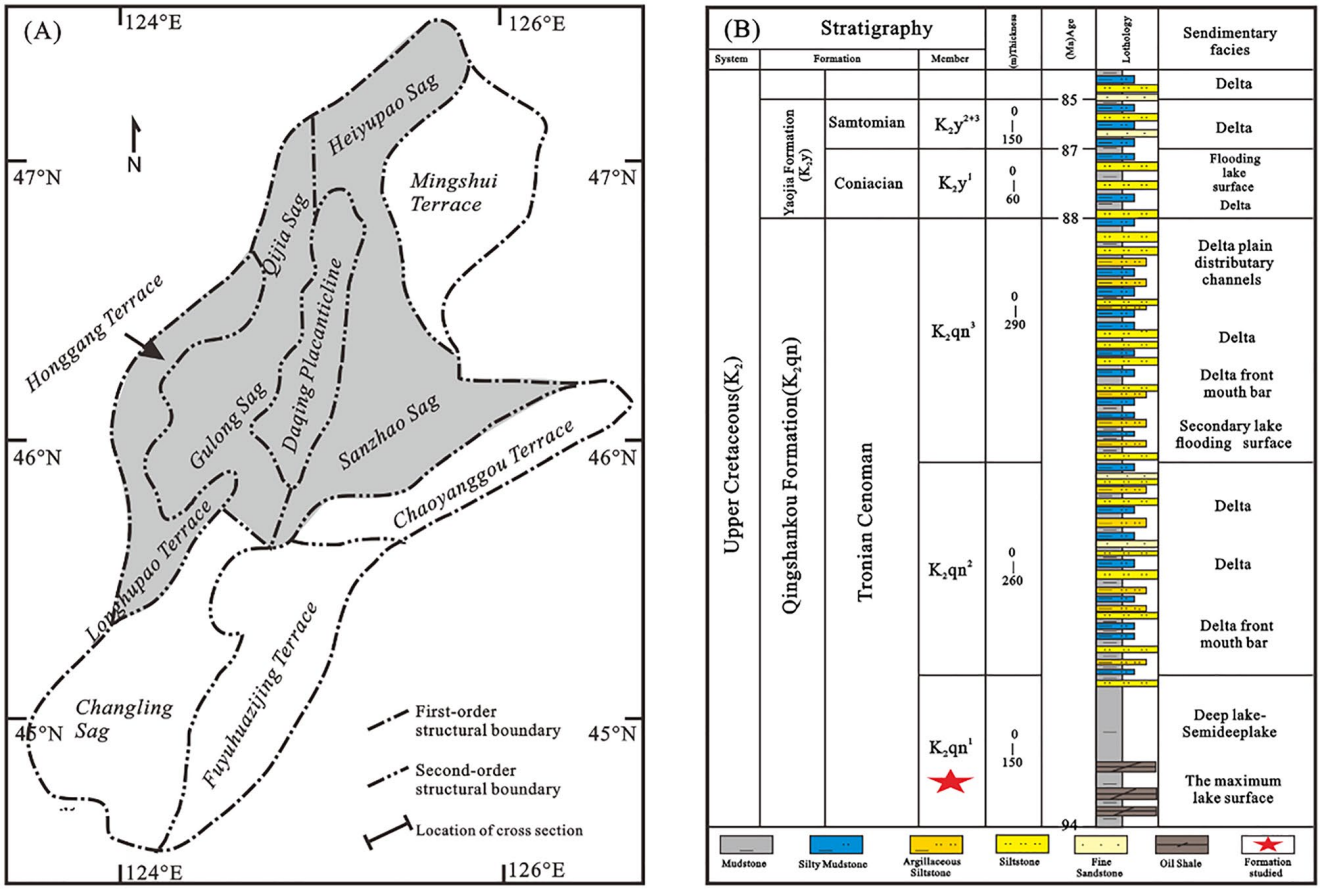
(**A**) Location map of the study area and (**B**) general stratigraphy of the Songliao Basin.

The Songliao Basin is located in northeast China between 119° 40′–128° 24′ E and 42° 25′–49° 23′ N. It is a NE–SW-trending basin, approximately 750 km long and 330–370 km wide, with an area of approximately 2.6 × 10^5 ^km^2^ (Fig. 1A). The Songliao Basin is surrounded by the Greater Khingan Mountains, Lesser Khingan Mountains, Zhangguangcai Mountains, and other hilly mountains and is connected to the sea through the southern Bohai Bay Basin. According to the regional tectonic characteristics, the basin is divided into six first-order tectonic units: northern dumping area, central depression area, northeast uplift area, southeast uplift area, southwest uplift area and western slope area^6,9^.

The Central Depression is the major target area for hydrocarbon exploration in the southern Songliao Basin^10^. The depression is characterised by Jurasssic to Cenezoic deposits (Fig. 1B), which include Cretaceous successions comprising strata such as the Mingshui, Sifangtai, Nenjiang, Yaojia, Qingshankou, Quantou, Denglouku, Yingcheng, and Shabezi formations. The oil shale of Nenjiang and Qingshankou formations were formed by the deposition of semi-deep to deep lake facies resulting from an increased subsidence rate which corresponded to a rapid expansion of the basin^7,8^. In particular, the Qingshankou oil shales were deposited during a rapid large transgression event. This resulted in the deposition of thick oil shale rich in organic matter that was terminated when the Songliao Basin experienced a regression and reduction in lake area^8^.

Several studies have emphasised the excellent shale oil reservoir potential of the Qingshankou Formation in the Changling sag of the central depression^3,4,10,11,15^**.** However, similar investigations conducted on Qingshankou shales of the Gulong sag could only reveal the implications of facies variations on shale oil generation^16^. Thus, large-scale shale oil production from Qingshankou shales of the Gulong sag^5,17^ necessitates further investigations into the mechanisms of mineral and organic matter evolution controlling the shale oil reservoir properties.

## Methods

### Mineral Composition and organic matter maturity

XRD analysis was conducted to determine the mineral composition of 202 cores obtained from three exploratory wells drilled into the first member of the Cretaceous Qingshankou Formation (K_2_qn_1_) within the Gulong sag of the Songliao Basin (Table S1). The weighed samples were crushed (< 200 mesh) and separated via the gravity method to ascertain the clay minerals. XRD analyses of both the separated clay and whole rock minerals were then conducted using Bruker XRD analyser (40 kV voltage and 30 mA current) using 2° to 70° scanning angles with a step of 0.02°. The depth and vitrinite reflectance (*Ro*) of the shale samples are provided in Table S2.

### Pore structure and types

The structures and types of pores in the shale samples were studied via FE-SEM. Prior to imaging, the bedding plane considerations in preparing samples included parallel cuts into 5 mm × 5 mm × 2 mm slices followed by polishing them perpendicularly with an argon ion polishing instrument (Ilion + II, Model 697, Gatan) for 6 to 8 h under an accelerating voltage of 6.0 kV. A gold film with a thickness of 10 nm was then deposited on the sample surface to increase the conductivity of the sample. FE-SEM were then conducted using a Zeiss SUPPA 55 instrument at an acceleration voltage of 10–20 keV, current of 20 nA, and working distance of 8–11 mm.

## Results

### Mineral composition

**Figure 2:**
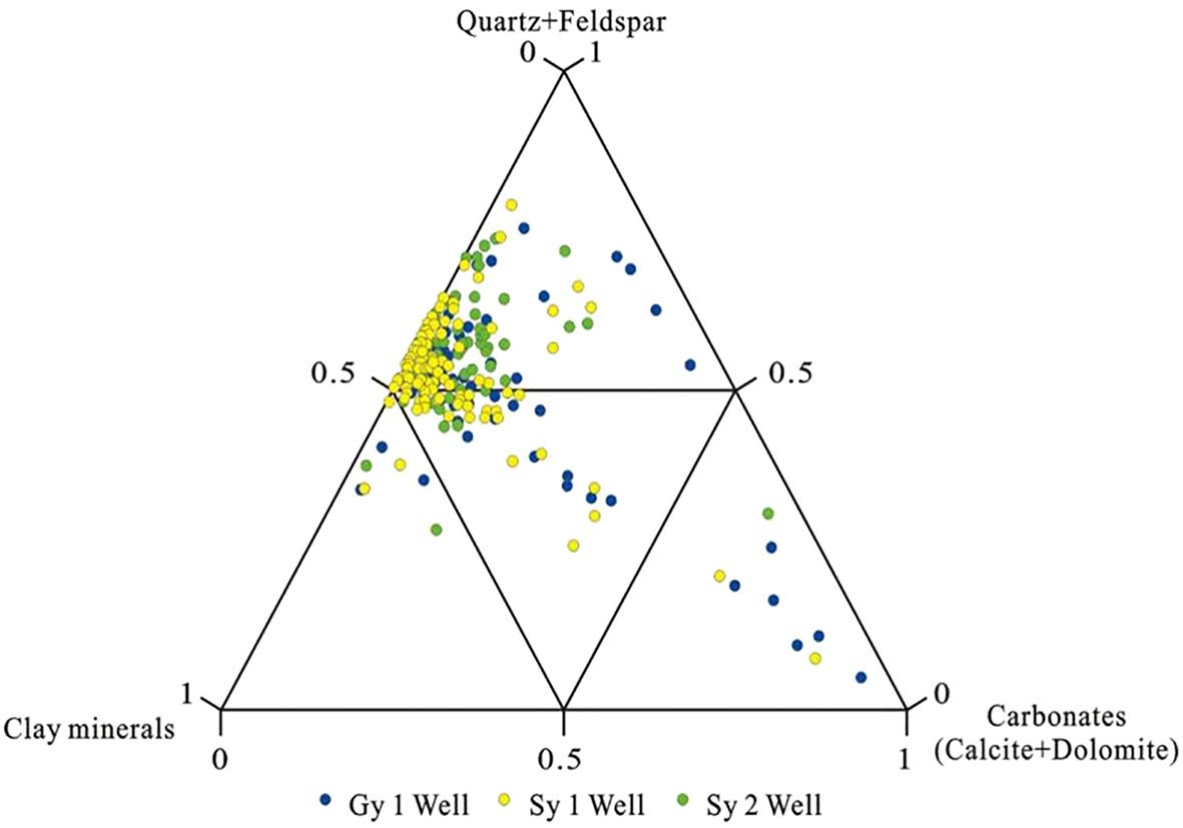
Ternary diagram of mineral compositions of the K_2_qn_1_ shales of the Songliao Basin.

A ternary diagram showing the relative proportions of total clay, clastic particles, and carbonate minerals illustrated the mineral composition of the fine-grained sedimentary rocks of K_2_qn_1_ (Fig. 2). This figure shows that most samples in K_2_qn_1_ were characterised by high quartz and feldspar contents. The dominant clay mineral was illite, followed by an illite–smectite mixed layer (I/S) and kaolinite. Carbonate minerals were concentrated in ostracum limestone.

The quartz content generally ranged from 4.7 to 41.4%, and some sections had slightly higher values of up to 30%. The K-feldspar content was relatively low, ranging from 0 to 24.1%, and the plagioclase content was relatively high, with average and maximum values of 17.24 and 28.8%, respectively. In the Gy 1 well, the average and highest quartz were 23.3 and 41.4%, respectively; the average and highest plagioclase contents were 15 and 23.3%, respectively. In the Sy 1 well, the average and highest quartz contents were 23.7 and 40.2%, respectively, and the average and highest plagioclase contents were 16.1 and 25.2%, respectively.

Carbonate minerals included calcite and dolomite. The average calcite content the in Sy 2 well was 0% and reached up to 8.26%, and the average iron dolomite content was 1.97%. In the Gy 1 well, the average and highest dolomite contents were 18.52 and 90.8%, respectively.

Clay minerals primarily comprised illite with an average content of 3.19%, followed by illite–smectite mixed layer clays with an average content of 0.55%. The kaolinite content was very low, and it was only found in a few layers.

### Pore observations from FE-SEM

Although studies have emplofyed low-pressure gas adsorption analysis to clarify the occurrence of intragranular and dissolution pores in the lacustrine Qingshankou shales of the Gulong sag^16^, the unreliability of low-pressure gas adsorption data for describing of shale pore types^18^ necessitates further investigations using sample imaging techniques such as FE-SEM. Furthermore, the relevance of using XRD and FE-SEM without combing with experimental fluid penetration techniques, such as mercury injection capillary pressure (MICP) and low-pressure gas adsorption, to evaluate the pore structure and composition in lacustrine shales was recently proposed in an investigation of pore structure in the Kongdian Formation^19^. The types and composition of pores within the lacustrine shale samples observed through FE-SEM are discussed in this section.

#### Intergranular pores

**Figure 3:**
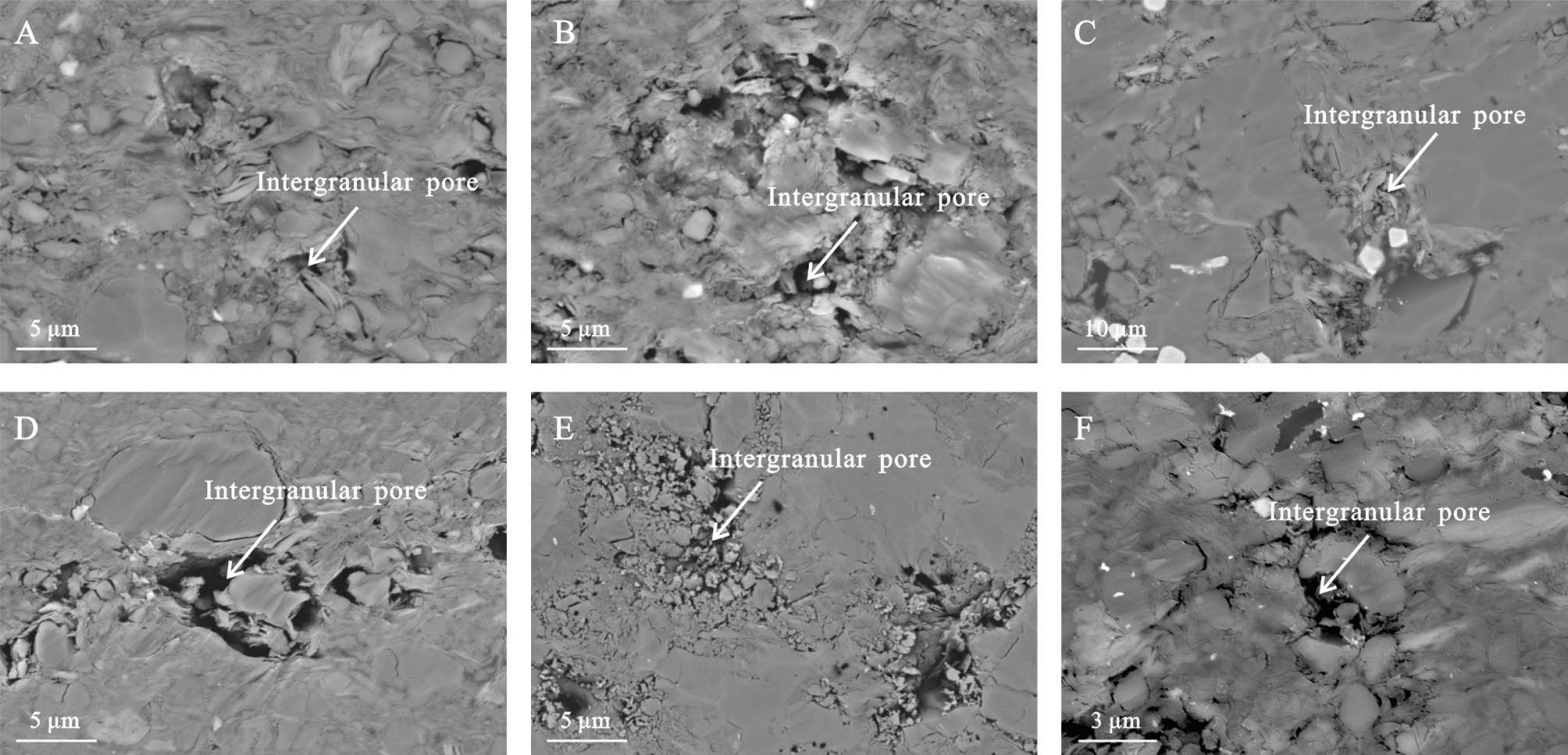
Intergranular pores of the K_2_qn_1_ shale in the Gulong sag.

The intergranular pores developed in the shale samples at the contact of mineral grains (Fig. 3) show polygonal and elongated shapes with few primary pores and primarily include silt- and muddy silt-laminae dominated by brittle minerals such as quartz and feldspar. The primary intergranular pore size was 1–20 μm.

Intergranular pores are primarily formed via a series of epigenetic transformations of clastic mineral particles, such as quartz, feldspar, and clay minerals, during compaction, cementation, and dissolution. Intergranular pores of quartz, feldspar, and other rigid particles have diverse morphologies, with a relatively large pore size. They differ from intergranular pores of clay particles, which are mostly long-axis shaped, distributed along the bedding plane, and have a pore size of 5–20 nm. This is because although pores between rigid particles such as quartz and feldspar are relatively undeveloped, plastic clay particles wrap rigid particles under compaction. However, owing to the compaction and dehydration of clay particles during rock formation, shrinkage cracks develop at the edges of rigid particles.

#### Intercrystal pores

**Figure 4:**
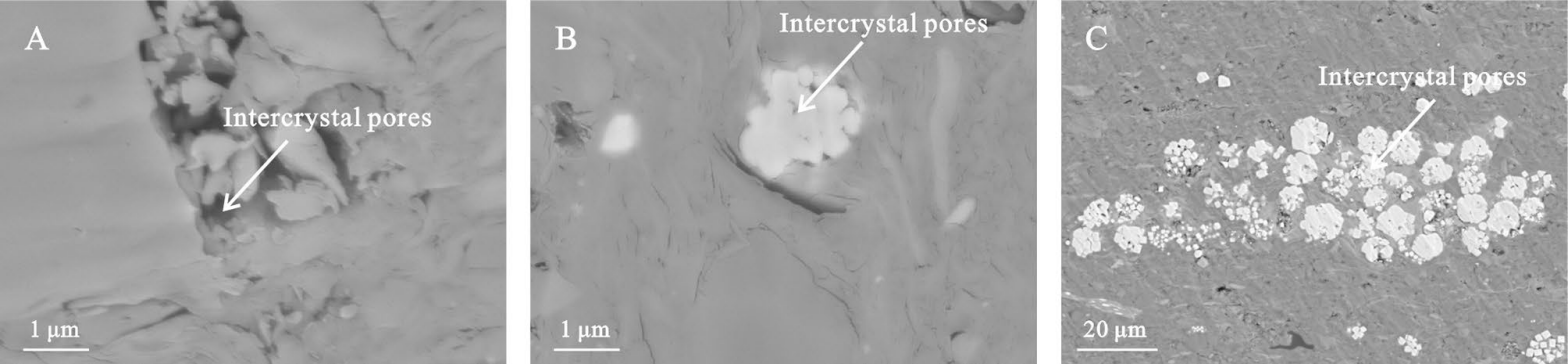
Intercrystal pores of the K_2_qn_1_ shale in the Gulong sag.

A small number of inter-crystal pores were observed in the skeletal minerals of the shale samples. These included quartz, feldspar, and microspherical granular/strawberry pyrite crystals which served as fillers (Fig. 4). The diameter of framboidal pyrite was approximately 1–10 μm and comprised pyrite crystals with a large number of intergranular pores between them, resulting in a pore diameter size of approximately 20–50 nm. Although the pores inside the framboidal body were well connected, the framboidal particles were relatively isolated.

#### Dissolution and organic matter pores

**Figure 5:**
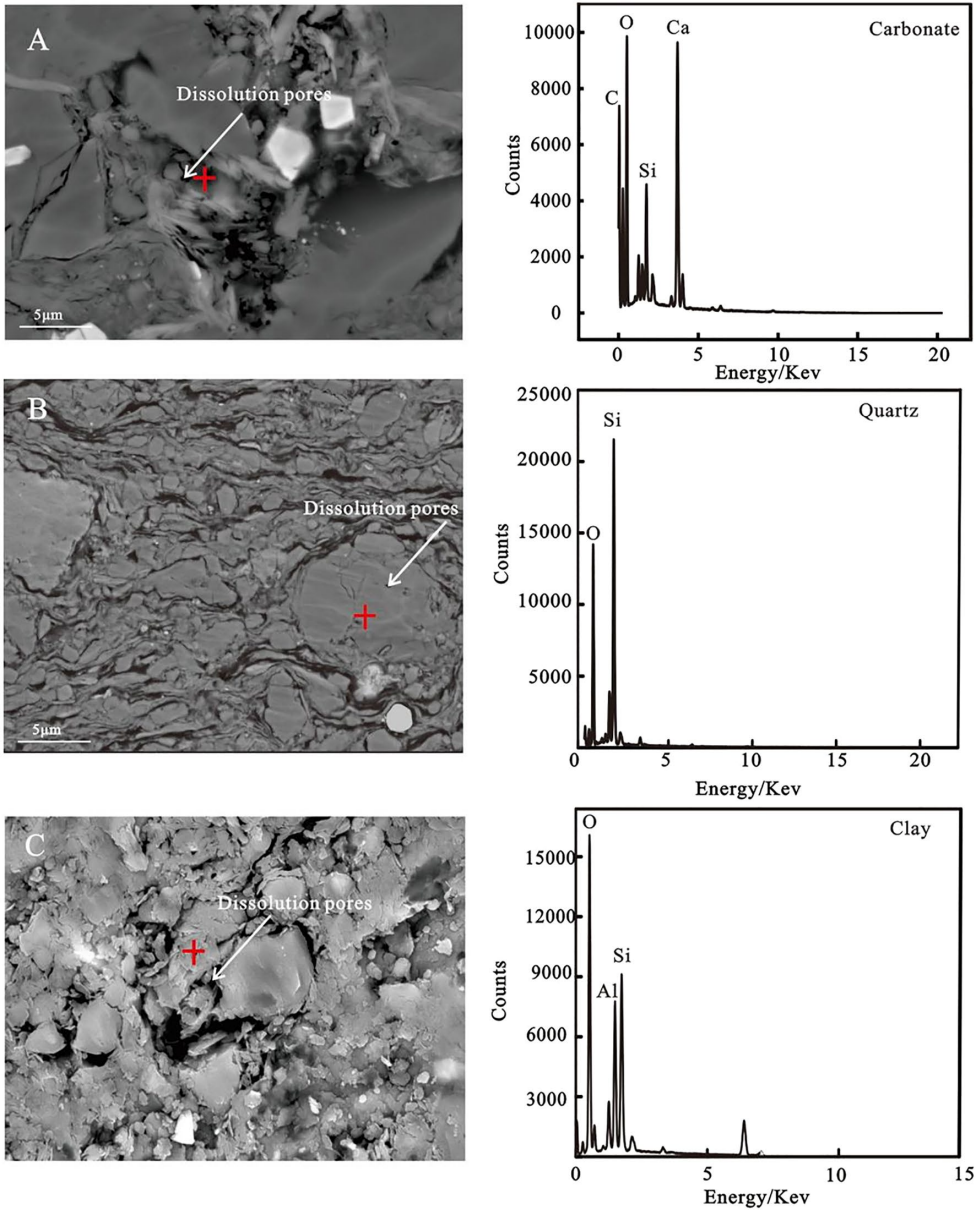
Pores of the K_2_qn_1_ shale in the Gulong sag derived from the dissolution of carbonate, quartz, and clay.

Figure 5 shows that the dissolution pores in the samples were derived from unstable minerals dissolved during deep burial. This can be demonstrated by the occurrence of pores in carbonate, quartz, and clay minerals which enhanced the pore connectivity in the samples. Their presence was probably attributed to the dissolution of these minerals during the thermal evolution of organic matter in the samples.

**Figure 6:**
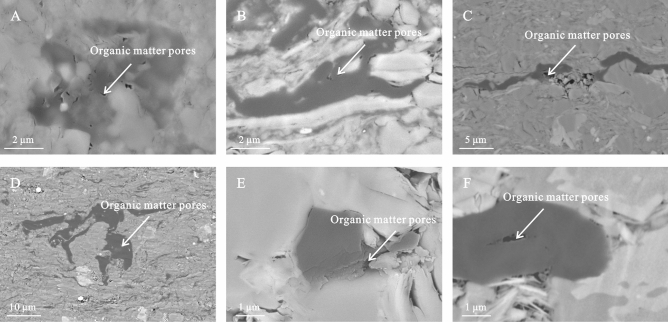
Organic matter pores of the K_2_qn_1_ shale in the Gulong sag.

During the hydrocarbon generation stage of thermal evolution, numerous pores are developed within the organic matter, which can be observed through SEM. Nanopores derived from the thermal evolution process are densely distributed within the organic matter, with an average pore size of 20 nm, which is significantly smaller than the intragranular and inter-crystal pores within the mineral matrix. The pore morphology was diverse, as both elliptical and elongated pores were developed, with pore diameters varying between 8 and 200 nm. Additionally, organic pores comprised both isolated individual pores and complex pore networks developed in sheets (Fig. 6). Notably, not all the organic matter developed pores (Fig. 6E).

## Discussion

### Effect of diagenesis on pores

**Figure 7:**
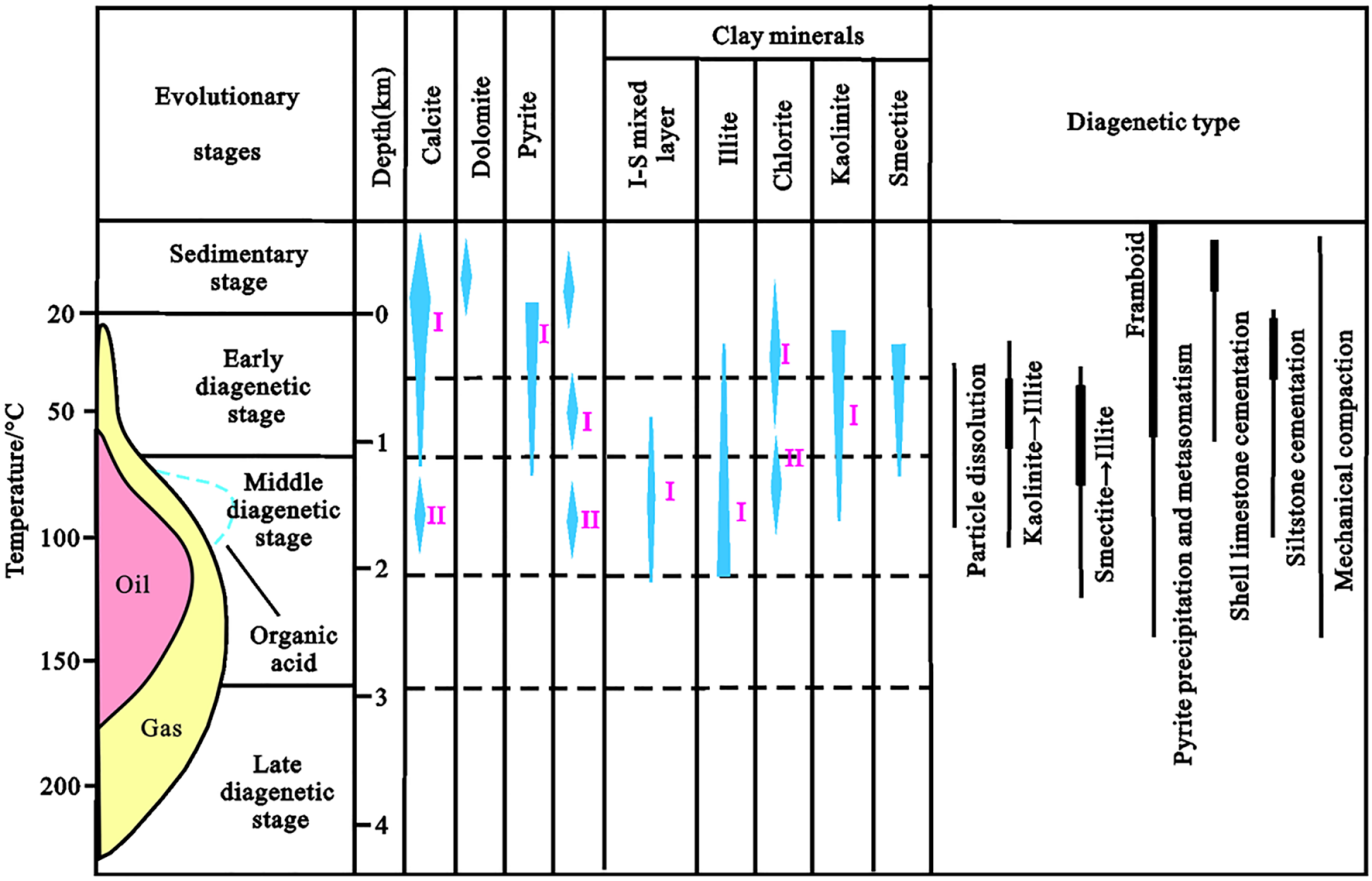
Diagenetic evolution of the K_2_qn_1_ shale in the Gulong sag.

By integrating the organic geochemical data (e.g. *R*_*o*_), transformation of clay mineral composition and diagenesis, a comprehensive division scheme was proposed for the diagenetic stages for the K_2_qn_1_ shales of the Gulong sag (Fig. 7). The shale in the Qingshankou Formation of the Qijia-Gulong sag was in the middle diagenetic stage.

During diagenesis, clay minerals, biogenic silica, organic matter, and carbonate were transformed. When the temperature was above 70 °C, the conversion of clay mineral composition was an important driving factor for the change in shale material, wherein the transformation of smectite or I/S into illite was predominant than that into I/S with high illite content at temperatures between 70 and 100 °C^20^. Furthermore, burial diagenesis results in the existence of silica as microcrystal quartz in the clay matrix that has undergone illitization. The transformation of biogenic silica during burial diagenesis is common in siliceous biogenic shales. It changes from opal-A to opal-CT and then becomes quartz^21^. Additionally, our study reveals that the crystal morphology of carbonate minerals changes during burial diagenesis, wherein the thermal evolution of organic matter greatly influences the crystal size and morphology of carbonate minerals^22^.

Thus, using observations from the backscattered electron (BSE) mode of FE-SEM, the diagenetic evolution sequence of the K_2_qn_1_ shale in the Gulong sag can be summarised as follows (Fig. 7): pyrite/siderite I → calcite I → chlorite I → dissolution I → authigenic quartz I/kaolinite I → illite–smectite mixed layer I → dissolution II → chlorite II → illite I/authigenic quartz II → calcite II. These observations correspond with a recent report stating that the transformation of swell clays to illite at the middle diagenetic stage makes the Gulong lacustrine shale more vulnerable to hydraulic fracturing^17^.

#### Syndiagenetic stage

Similar to other clastic rocks, argillaceous sediments were unconsolidated after deposition in a soft mud state, thereby developing primary pores and free water. As the sediments in this stage were not separated from the overlying water, the pore water retained the properties of the bottom water of the sedimentary lake basin, which was rich in metal cations, such as Fe^2+^, Mg^2+^, Ca^2+^ and Na^+^. In this anoxic environment, self-shaped microcrystalline siderite, framboidal pyrite aggregates, and a small amount of micritic microcrystal calcite were formed. The development of primary pores in this stage provided sufficient space for the development of siderite and pyrite cements (Fig. 4). Thus, the degree of self-shape was high, crystals were large, scattered distribution of self-shape single pyrite crystal reached 0.01 μm, and the long axis of pyrite aggregate and siderite crystals reached 0.05 μm^23^.

#### Early diagenetic stage

With the gravity load effect of overlying water and deposits, the enriched free water in the primary porosity continuously decrease; the primary porosity sharply decreases, and the slime sediments gradually change from unconsolidated sediments to weakly consolidation–semi-consolidation^24^. At this stage, small amounts of pyrite and siderite continued to form. Furthermore, in the Fe^2+^- and Mg^2+^-rich alkaline diagenetic environment, chlorite cement began to form, and chlorite films were formed along the surface of clastic particles in argillaceous rocks with a high silt content^25^. At this stage, with increasing burial depth, temperature and pressure, as a result of continuous cementation and significant compaction, plastic clay was continuously deformed, broken and rearranged, and rocks were almost consolidated. The diagenetic fluid environment gradually changed from alkaline to acidic, where unstable feldspar, carbonate and other easily soluble minerals were corroded forming secondary dissolution pores (Fig. 5) because CO_2_ and organic acids entered the pore fluid and generated hydrocarbons with thermal evolution. The K^+^, Ca^2+^, Al^3+^ and Si^4+^ contents in the pore fluid continuously increased, forming authigenic quartz and kaolinite cement that filled the intergranular and feldspar dissolution pores after the dissolution of feldspar. During this stage, with temperatures ranging from 35 to 70 °C, abundant smectite in argillaceous sedimentary rocks gradually began transforming to illite, thereby forming an intermediate product, namely, the illite–smectite mixed layer^20^.

#### Middle diagenetic stage

In the middle diagenetic stage, the K_2_qn_1_ shales in the Gulong sag were completely consolidated, the formation temperature reached 85–140 °C^20^, and a large amount of smectite transformed to illite. Under the catalytic activity of temperature and clay minerals, the organic matter evolution entered the thermal catalytic hydrocarbon generation stage, forming a large number of carboxylic acids and dissolving in water, making the diagenetic fluid weakly acidic. Thus, feldspar and carbonate minerals underwent continuous dissolution. However, the water rock reaction was not significant; thus, the pores formed by dissolution were also limited because the shale was subjected to continuous high compaction and cementation^26,27^. Owing to the continuous hydrocarbon generation and expulsion, most organic acids are constantly discharged from shale reservoirs^28^, and acidic substances in pore fluids are consumed during dissolution. The diagenetic fluid environment thus gradually changed from acidic to weakly alkaline owing to the decarboxylation of carboxylic acids. In an alkaline diagenetic environment, a small amount of hairy authigenic illite, amorphous microcrystalline quartz, leucine calcite and iron dolomite cement filled some residual intergranular pores and secondary dissolution pores. Detrital quartz particles underwent weak alkaline dissolution, forming a small amount of quartz dissolution pores (Fig. 5).

### Pore evolution of shale

**Figure 8:**
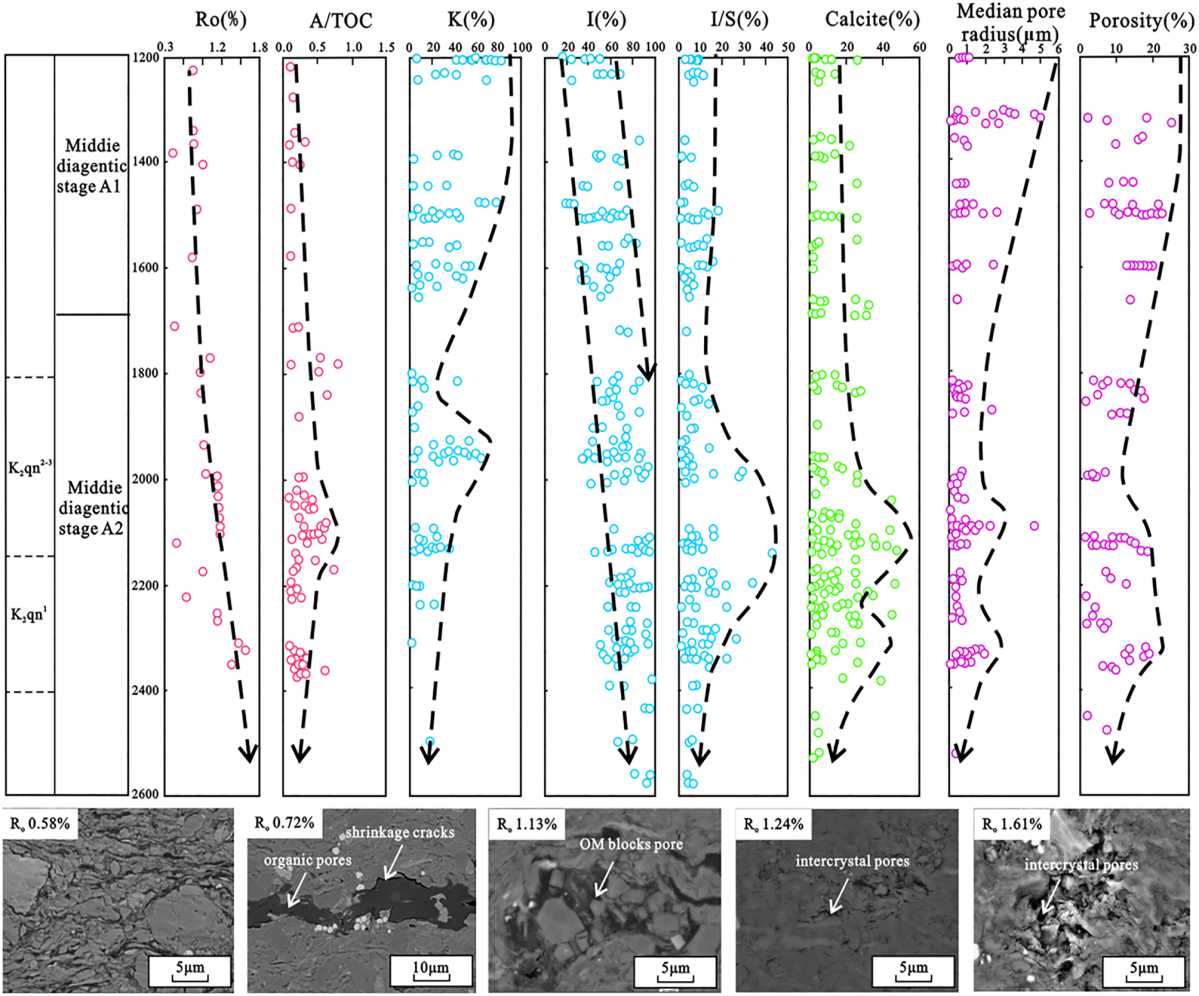
Shale pore evolution of the K_2_qn_1_ shale in the Gulong sag.

Reservoirs at different diagenetic stages have different diagenetic strengths and physical properties during burial diagenesis^29,30^. The porosity of reservoir rocks is affected by both burial depth and time. The burial time has a continuous effect on the porosity, and the uplift of the stratum decreases the effect of depth^31^. The K_2_qn_1_ shale in the study area reached its maximum burial depth in the early Late Cretaceous (100 Ma), followed by large-scale uplift. After the Palaeogene, slight subsidence occurred again; however the burial depth did not exceed the maximum burial depth during the early Late Cretaceous; therefore, the depth effect disappeared during the maximum burial depth period to the present. Combining the differences in diagenesis, thermal evolution, hydrocarbon generation, and tectonics during the different burial stages, the pore evolution of the K_2_qn_1_ shale can be divided into two sub-stages (Fig. 8).

In the middle A1 stage, wherein the rate of porosity decrease was relatively low, the porosity decreased from 25 to 10% (Fig. 8). The burial depth of this stage was 1000–1700 m; the thermal evolution of organic matter began and gradually entered the peak of hydrocarbon generation, as shown by the increase in the *Ro* value with depth. Furthermore, at this stage, the porosity significantly decreased as the drainage of pore water became difficult and the intensity of compaction gradually weakened. With the progress of thermal evolution and hydrocarbon generation, organic matter gradually began to crack and formed pores. Simultaneously, large amounts of organic acids were produced during hydrocarbon generation, causing the corrosion of feldspar, carbonate and other susceptible minerals and the formation of secondary dissolution pores. Additionally, the mineral conversion of smectite and kaolinite to chlorite reduced the development of inter-crystal micropores in clay minerals. Owing to the conversion of smectite to illite, authigenic quartz cemented the micropores; the conversion of potash feldspar to hydronic feldspar inhibited the dissolution of potash feldspar and acidic plagioclase at this stage, thereby reducing the development of secondary dissolution pores.

In the middle A2 stage, the porosity slowly decreased until it became stable, and the porosity decreased from 10 to 5%. At this stage, the burial depth was > 1700 m, and the diagenetic fluid environment gradually changed from acidic to alkaline. Compaction during the diagenesis was not evident at this stage, and cementation was enhanced. In the early stage, as the smectite transformed to illite, a large amount of authigenic quartz had cemented the pores as the diagenetic environment changed from acidic to alkaline, and calcite and iron dolomite cement filled the pores. The dissolution was relatively weak at this stage, and only a portion of the clay minerals and quartz underwent dissolution to form a small number of secondary dissolution pores.

## Conclusions

In this study, organic matter enrichment and mineral and pores evolutions in the K_2_qn_1_ lacustrine shales of the Gulong sag were experimentally evaluated to resolve the shale oil production problems in the lacustrine reservoirs of the petroliferous Songliao Basin. Organic and inorganic pore types were observed, with the latter being influenced by diagenetic processes, such as compaction, pressure solution, cementation, and dissolution. Compaction, pressure solution, and cementation were the dominant factors that destroyed the pore structure of shale, whereas dissolution positively influenced the porosity. Notably, the K_2_qn_1_ shale in the Gulong sag is also observed to be in the middle diagenetic stage A (*R*_*o*_ = 0.5–1.3%), wherein the kaolinite occurring in combination with mixed layered clays was replaced by illite with increasing depth. Furthermore, two sub-stages can be characterized within the oil-window maturity. In the middle A1 stage, the rate of porosity decrease was relatively low, and in the middle A2 stage, the porosity slowly decreased until reaching stablility. These observations are significant for shale oil exploration in petroliferous lacustrine shale oil reservoirs worldwide.

## Supplementary Information

Supplementary Information 1.

Supplementary Information 2.

## Data Availability

The data generated during and/or analyzed during the current study are available from the corresponding author on reasonable request.
