# Supplementary material for: Pore types, genesis, and evolution model of lacustrine oil-prone shale: a case study of the Cretaceous Qingshankou Formation, Songliao Basin, NE China
Source: Sci Rep. 2022 Oct 14;12:17210. doi: 10.1038/s41598-022-21154-y (PMC9568562; doi:10.1038/s41598-022-21154-y)
Supplement: Supplementary file 1 — Supplementary Information 1. [file 41598_2022_21154_MOESM1_ESM.docx]

| Table S1. Mineralogical composition of shale samples | | | | | | | |
| --- | --- | --- | --- | --- | --- | --- | --- |
| Sample | Mineral composition (%) | | | | | | |
| NO. | Quartz | K-feldspar | Plagioclase | Calcite | Dolomite | Pyrite | Clay |
| Gy1-1 | 34.9 | 15.6 | 20.6 | 1.1 | 5.3 | 4.3 | 18.2 |
| Gy1-2 | 32.5 | 13.0 | 22.1 | 0.4 | 2.1 | 2.1 | 27.9 |
| Gy1-3 | 39.8 | 5.6 | 21.1 | 1.1 | 3.2 | 3.7 | 25.5 |
| Gy1-4 | 41.4 | 7.3 | 18.8 | 1.4 | 20.9 | 3.4 | 6.8 |
| Gy1-5 | 39.8 | 5.9 | 20.1 | 1.1 | 24.1 | 3.1 | 5.8 |
| Gy1-6 | 20.9 | 9.0 | 21.3 | 12.3 | 29.1 | 2.8 | 4.6 |
| Gy1-7 | 18.7 | 5.5 | 7.3 | 15.6 | 24.8 | 1.2 | 26.8 |
| Gy1-8 | 18.1 | 4.0 | 6.4 | 18.1 | 19.3 | 4.6 | 29.5 |
| Gy1-9 | 25.2 | 2.6 | 3.5 | 5.5 | 27.4 | 3.7 | 32.0 |
| Gy1-10 | 19.8 | 0.7 | 15.9 | 13.1 | 19.1 | 0.2 | 31.2 |
| Gy1-11 | 12.5 | 0.4 | 4.2 | 5.5 | 66.5 | 0.1 | 10.9 |
| Gy1-12 | 6.2 | 0.8 | 2.4 | 0.0 | 79.0 | 0.7 | 10.9 |
| Gy1-13 | 5.9 | 0.3 | 3.5 | 13.1 | 68.3 | 1.8 | 7.1 |
| Gy1-14 | 5.1 | 0.0 | 0.0 | 0.0 | 90.8 | 0.0 | 4.1 |
| Gy1-15 | 23.7 | 0.3 | 9.7 | 3.1 | 0.0 | 0.8 | 62.4 |
| Gy1-16 | 21.2 | 6.6 | 5.6 | 5.2 | 6.4 | 2.5 | 52.4 |
| Gy1-17 | 27.8 | 0.6 | 12.3 | 2.9 | 0.0 | 0.4 | 56.0 |
| Gy1-18 | 26.5 | 0.9 | 16.9 | 0.6 | 2.3 | 5.3 | 47.5 |
| Gy1-19 | 17.6 | 5.0 | 19.9 | 2.3 | 5.4 | 5.9 | 43.9 |
| Gy1-20 | 15.4 | 8.7 | 19.4 | 1.3 | 8.9 | 4.2 | 42.1 |
| Gy1-21 | 26.6 | 1.2 | 16.6 | 1.1 | 11.1 | 3.5 | 39.9 |
| Gy1-22 | 22.1 | 3.4 | 18.1 | 1.4 | 10.6 | 1.5 | 43.0 |
| Gy1-23 | 14.8 | 6.9 | 19.4 | 2.3 | 12.3 | 1.6 | 42.7 |
| Gy1-24 | 24.5 | 3.6 | 20.3 | 0.0 | 5.8 | 2.8 | 43.0 |
| Gy1-25 | 17.7 | 8.4 | 21.1 | 0.7 | 4.3 | 5.0 | 42.8 |
| Gy1-26 | 16.0 | 10.8 | 22.7 | 2.4 | 3.5 | 3.1 | 41.5 |
| Gy1-27 | 15.9 | 10.0 | 21.4 | 0.7 | 7.2 | 4.3 | 40.5 |
| Gy1-28 | 33.5 | 0.7 | 17.6 | 3.6 | 5.8 | 0.2 | 38.6 |
| Gy1-29 | 29.8 | 2.5 | 15.2 | 7.8 | 3.3 | 3.1 | 38.3 |
| Gy1-30 | 27.8 | 4.1 | 19.1 | 2.1 | 3.3 | 4.2 | 39.3 |
| Gy1-31 | 15.5 | 15.3 | 20.6 | 0.0 | 6.5 | 4.5 | 37.6 |
| Gy1-32 | 30.5 | 4.8 | 16.8 | 0.5 | 3.9 | 4.1 | 39.3 |
| Gy1-33 | 31.2 | 3.6 | 19.3 | 0.8 | 2.2 | 2.3 | 40.6 |
| Gy1-34 | 28.6 | 10.5 | 17.4 | 0.0 | 3.1 | 2.6 | 37.8 |
| Gy1-35 | 26.5 | 6.6 | 21.1 | 1.4 | 4.2 | 4.2 | 36.0 |
| Gy1-36 | 33.3 | 2.8 | 23.3 | 0.0 | 2.3 | 2.4 | 36.0 |
| Gy1-37 | 21.7 | 13.5 | 19.3 | 0.1 | 5.9 | 5.4 | 34.1 |
| Gy1-38 | 17.8 | 13.6 | 23.2 | 0.9 | 7.3 | 6.4 | 30.8 |
| Gy1-39 | 14.4 | 14.5 | 19.5 | 3.0 | 9.2 | 5.8 | 33.6 |
| Gy1-40 | 28.9 | 3.7 | 18.8 | 1.9 | 15.2 | 0.5 | 30.9 |
| Gy1-41 | 26.2 | 13.0 | 20.7 | 0.0 | 14.7 | 4.9 | 20.6 |
| Gy1-42 | 26.7 | 6.3 | 9.2 | 0.7 | 16.4 | 3.4 | 37.3 |
| Gy1-43 | 27.8 | 1.2 | 19.3 | 2.1 | 13.2 | 0.8 | 35.5 |
| Gy1-44 | 25.8 | 5.7 | 11.9 | 3.4 | 15.4 | 4.2 | 33.6 |
| Gy1-45 | 31.7 | 0.5 | 14.5 | 3.1 | 20.0 | 0.2 | 30.0 |
| Gy1-46 | 24.2 | 4.6 | 9.6 | 7.0 | 18.9 | 1.2 | 34.5 |
| Gy1-47 | 11.1 | 3.0 | 2.3 | 9.3 | 55.9 | 3.1 | 15.4 |
| Gy1-48 | 12.7 | 2.7 | 8.8 | 6.3 | 61.2 | 1.2 | 7.0 |
| Gy1-49 | 23.2 | 0.7 | 1.2 | 5.2 | 62.3 | 0.3 | 7.0 |
| Sy1-1 | 21.2 | 0.1 | 12.9 | 1.1 | 2.5 | 0.4 | 61.8 |
| Sy1-2 | 20.7 | 1.5 | 15.8 | 2.0 | 4.9 | 0.3 | 54.7 |
| Sy1-3 | 29.1 | 0.6 | 17.2 | 0.5 | 0.0 | 1.2 | 51.4 |
| Sy1-4 | 29.3 | 0.5 | 20.3 | 0.0 | 0.0 | 0.3 | 49.6 |
| Sy1-5 | 29.6 | 5.8 | 13.8 | 0.0 | 0.0 | 2.4 | 48.4 |
| Sy1-6 | 20.9 | 8.8 | 14.6 | 0.5 | 1.8 | 4.5 | 48.9 |
| Sy1-7 | 23.2 | 4.8 | 18.2 | 0.3 | 2.0 | 3.5 | 48.1 |
| Sy1-8 | 23.9 | 5.4 | 18.7 | 1.8 | 0.0 | 2.4 | 47.8 |
| Sy1-9 | 26.7 | 4.4 | 16.5 | 0.1 | 0.2 | 4.6 | 47.5 |
| Sy1-10 | 22.3 | 9.5 | 16.2 | 0.3 | 0.5 | 4.4 | 46.8 |
| Sy1-11 | 21.7 | 7.8 | 17.7 | 1.4 | 0.0 | 4.7 | 46.7 |
| Sy1-12 | 22.3 | 10.1 | 13.9 | 0.7 | 2.4 | 4.8 | 45.8 |
| Sy1-13 | 19.0 | 12.0 | 15.8 | 1.7 | 2.1 | 3.9 | 45.5 |
| Sy1-14 | 18.6 | 12.7 | 16.7 | 1.6 | 1.7 | 3.7 | 45.1 |
| Sy1-15 | 23.4 | 2.8 | 17.4 | 2.0 | 3.1 | 3.4 | 47.9 |
| Sy1-16 | 15.5 | 5.4 | 25.2 | 1.5 | 3.2 | 2.1 | 47.1 |
| Sy1-17 | 26.3 | 3.7 | 17.3 | 1.2 | 4.2 | 1.3 | 46.0 |
| Sy1-18 | 27.7 | 0.8 | 18.2 | 1.1 | 4.9 | 0.7 | 46.6 |
| Sy1-19 | 26.3 | 2.1 | 20.1 | 1.7 | 5.7 | 0.2 | 43.9 |
| Sy1-20 | 22.2 | 6.9 | 15.4 | 1.4 | 5.9 | 4.1 | 44.1 |
| Sy1-21 | 22.1 | 8.3 | 14.9 | 2.4 | 2.4 | 4.5 | 45.4 |
| Sy1-22 | 24.1 | 7.4 | 13.8 | 2.4 | 3.4 | 4.8 | 44.1 |
| Sy1-23 | 23.2 | 8.3 | 14.7 | 1.7 | 2.9 | 4.9 | 44.3 |
| Sy1-24 | 19.8 | 12.2 | 15.9 | 1.3 | 4.1 | 3.5 | 43.2 |
| Sy1-25 | 25.7 | 3.0 | 15.2 | 1.2 | 9.0 | 2.1 | 43.8 |
| Sy1-26 | 27.2 | 3.0 | 16.8 | 1.5 | 8.7 | 2.3 | 40.5 |
| Sy1-27 | 24.6 | 7.1 | 13.5 | 3.7 | 7.8 | 4.1 | 39.2 |
| Sy1-28 | 31.2 | 2.5 | 13.4 | 2.2 | 8.4 | 1.3 | 41.0 |
| Sy1-29 | 24.5 | 4.1 | 15.9 | 1.7 | 9.0 | 3.2 | 41.6 |
| Sy1-30 | 22.6 | 4.2 | 17.2 | 1.2 | 11.0 | 3.5 | 40.3 |
| Sy1-31 | 15.9 | 8.3 | 16.6 | 0.3 | 13.0 | 5.0 | 40.9 |
| Sy1-32 | 28.7 | 0.5 | 15.8 | 3.1 | 12.5 | 0.7 | 38.7 |
| Sy1-33 | 25.6 | 4.6 | 14.2 | 2.0 | 13.4 | 2.4 | 37.9 |
| Sy1-34 | 19.7 | 7.1 | 15.2 | 4.3 | 12.4 | 4.8 | 36.5 |
| Sy1-35 | 23.6 | 3.8 | 16.8 | 6.0 | 11.5 | 1.5 | 36.8 |
| Sy1-36 | 27.9 | 4.1 | 15.1 | 5.7 | 13.2 | 2.1 | 31.9 |
| Sy1-37 | 20.4 | 7.5 | 18.2 | 4.5 | 12.4 | 3.5 | 33.5 |
| Sy1-38 | 21.3 | 7.0 | 18.7 | 6.4 | 7.0 | 4.2 | 35.4 |
| Sy1-39 | 16.5 | 9.4 | 21.8 | 2.9 | 9.0 | 3.8 | 36.6 |
| Sy1-40 | 17.2 | 9.2 | 19.7 | 2.4 | 5.4 | 4.8 | 41.3 |
| Sy1-41 | 21.2 | 11.8 | 14.2 | 2.5 | 4.3 | 4.5 | 41.5 |
| Sy1-42 | 23.4 | 10.3 | 15.4 | 2.4 | 2.7 | 3.8 | 42.0 |
| Sy1-43 | 23.7 | 10.6 | 14.9 | 1.5 | 4.7 | 4.7 | 39.9 |
| Sy1-44 | 20.6 | 15.5 | 13.4 | 1.3 | 3.8 | 5.0 | 40.4 |
| Sy1-45 | 27.8 | 5.0 | 18.4 | 1.4 | 2.4 | 3.2 | 41.8 |
| Sy1-46 | 19.5 | 13.6 | 16.8 | 1.1 | 2.5 | 4.8 | 41.7 |
| Sy1-47 | 24.6 | 10.1 | 16.4 | 1.2 | 1.9 | 4.2 | 41.6 |
| Sy1-48 | 19.9 | 15.5 | 14.3 | 1.1 | 2.8 | 3.7 | 42.7 |
| Sy1-49 | 16.4 | 17.4 | 15.9 | 0.3 | 3.0 | 3.9 | 43.1 |
| Sy1-50 | 29.7 | 7.0 | 14.6 | 0.5 | 2.4 | 2.1 | 43.7 |
| Sy1-51 | 31.2 | 4.1 | 16.7 | 2.1 | 0.0 | 1.3 | 44.6 |
| Sy1-52 | 27.9 | 5.3 | 17.8 | 0.5 | 0.0 | 2.4 | 46.1 |
| Sy1-53 | 15.8 | 16.2 | 17.5 | 0.4 | 0.0 | 4.5 | 45.6 |
| Sy1-54 | 23.4 | 12.6 | 13.3 | 0.7 | 0.0 | 3.8 | 46.2 |
| Sy1-55 | 25.6 | 8.4 | 15.8 | 0.0 | 0.0 | 4.9 | 45.4 |
| Sy1-56 | 18.8 | 16.4 | 16.5 | 0.0 | 0.0 | 3.5 | 44.9 |
| Sy1-57 | 23.9 | 10.1 | 16.3 | 1.9 | 0.0 | 4.2 | 43.7 |
| Sy1-58 | 24.1 | 12.7 | 13.5 | 0.9 | 1.9 | 4.3 | 42.6 |
| Sy1-59 | 21.6 | 15.2 | 15.8 | 0.3 | 1.0 | 3.5 | 42.6 |
| Sy1-60 | 20.8 | 15.3 | 16.7 | 0.4 | 0.0 | 3.3 | 43.5 |
| Sy1-61 | 23.1 | 12.2 | 17.2 | 0.0 | 0.0 | 3.6 | 43.9 |
| Sy1-62 | 22.2 | 16.4 | 15.6 | 0.0 | 0.0 | 2.9 | 42.9 |
| Sy1-63 | 26.5 | 9.9 | 16.9 | 0.5 | 0.0 | 4.1 | 42.1 |
| Sy1-64 | 24.9 | 14.1 | 16.4 | 0.5 | 1.0 | 1.8 | 41.4 |
| Sy1-65 | 18.5 | 21.5 | 16.5 | 0.0 | 0.0 | 1.9 | 41.5 |
| Sy1-66 | 21.9 | 20.7 | 13.7 | 1.0 | 0.0 | 2.4 | 40.3 |
| Sy1-67 | 22.8 | 17.1 | 16.8 | 0.3 | 0.0 | 2.7 | 40.3 |
| Sy1-68 | 20.6 | 19.5 | 15.8 | (0.0) | 0.0 | 3.5 | 40.6 |
| Sy1-69 | 30.4 | 8.2 | 16.9 | 0.3 | 0.7 | 4.8 | 38.8 |
| Sy1-70 | 19.1 | 21.6 | 15.7 | 0.0 | 0.0 | 4.1 | 39.7 |
| Sy1-71 | 20.5 | 20.3 | 16.4 | 0.0 | 0.0 | 4.3 | 38.4 |
| Sy1-72 | 35.6 | 1.9 | 17.9 | 1.4 | 1.2 | 3.5 | 38.5 |
| Sy1-73 | 23.1 | 15.1 | 15.4 | 1.4 | 4.9 | 3.2 | 36.9 |
| Sy1-74 | 25.6 | 16.4 | 13.8 | 1.4 | 3.1 | 4.5 | 35.2 |
| Sy1-75 | 28.7 | 10.5 | 16.9 | 0.5 | 1.8 | 4.7 | 36.9 |
| Sy1-76 | 27.9 | 15.5 | 15.8 | 0.4 | 0.0 | 3.8 | 36.6 |
| Sy1-77 | 24.6 | 16.1 | 20.6 | 0.2 | 0.0 | 3.2 | 35.3 |
| Sy1-78 | 27.8 | 14.2 | 18.7 | 1.9 | 0.0 | 3.1 | 34.2 |
| Sy1-79 | 35.7 | 9.1 | 15.2 | 0.9 | 1.5 | 2.8 | 34.7 |
| Sy1-80 | 23.4 | 0.1 | 15.4 | 0.5 | 22.5 | 0.0 | 38.0 |
| Sy1-81 | 21.8 | 1.0 | 16.3 | 1.9 | 24.7 | 1.0 | 33.3 |
| Sy1-82 | 19.9 | 12.5 | 19.9 | 4.2 | 15.9 | 4.3 | 23.3 |
| Sy1-83 | 26.5 | 6.9 | 24.2 | 2.1 | 7.5 | 2.1 | 30.7 |
| Sy1-84 | 30.7 | 8.7 | 19.5 | 2.1 | 15.2 | 3.5 | 20.3 |
| Sy1-85 | 36.5 | 8.2 | 18.1 | 2.5 | 16.5 | 3.4 | 14.8 |
| Sy1-86 | 39.3 | 7.9 | 16.6 | 0.7 | 2.9 | 3.8 | 28.7 |
| Sy1-87 | 38.5 | 9.9 | 18.4 | 0.7 | 0.0 | 2.7 | 29.8 |
| Sy1-88 | 39.8 | 13.7 | 15.8 | 1.0 | 2.7 | 4.6 | 22.3 |
| Sy1-89 | 40.2 | 9.6 | 24.4 | 2.9 | 0.0 | 4.8 | 18.2 |
| Sy1-90 | 17.8 | 24.1 | 16.8 | 1.7 | 20.8 | 4.3 | 14.6 |
| Sy1-91 | 16.2 | 0.7 | 15.4 | 1.3 | 35.7 | 2.5 | 28.2 |
| Sy1-92 | 18.5 | 1.8 | 8.8 | 0.9 | 38.4 | 1.2 | 30.4 |
| Sy1-93 | 12.7 | 0.1 | 11.5 | 2.8 | 35.7 | 1.4 | 35.8 |
| Sy1-94 | 12.2 | 0.3 | 7.9 | 3.5 | 58.7 | 0.5 | 16.9 |
| Sy1-95 | 4.7 | 0.1 | 3.3 | 7.1 | 75.5 | 0.0 | 9.3 |
| Sy1-96 | 5.4 | 0.2 | 2.2 | 5.2 | 77.4 | 0.3 | 9.3 |
| Sy2-1 | 22.5 | 1.1 | 3.5 | 3.6 | 13.6 | 1.2 | 54.5 |
| Sy2-2 | 17.8 | 0.9 | 19.0 | 1.9 | 0.0 | 0.6 | 59.7 |
| Sy2-3 | 20.5 | 0.3 | 20.3 | 2.8 | 9.4 | 3.4 | 43.3 |
| Sy2-4 | 25.7 | 1.3 | 14.1 | 3.2 | 7.1 | 3.2 | 45.4 |
| Sy2-5 | 25.9 | 3.5 | 14.6 | 0.3 | 8.1 | 2.9 | 44.7 |
| Sy2-6 | 20.5 | 6.9 | 18.7 | 2.9 | 4.1 | 1.8 | 45.1 |
| Sy2-7 | 19.8 | 6.8 | 18.1 | 1.2 | 3.8 | 4.5 | 45.8 |
| Sy2-8 | 23.4 | 0.0 | 21.3 | 0.8 | 1.7 | 3.5 | 49.3 |
| Sy2-9 | 21.6 | 10.2 | 14.0 | 1.2 | 7.7 | 2.9 | 42.3 |
| Sy2-10 | 25.9 | 5.7 | 15.7 | 4.5 | 5.4 | 2.7 | 40.1 |
| Sy2-11 | 27.7 | 0.3 | 18.9 | 3.5 | 10.1 | 3.1 | 36.4 |
| Sy2-12 | 22.9 | 6.1 | 17.9 | 4.9 | 10.8 | 4.6 | 32.8 |
| Sy2-13 | 20.1 | 6.2 | 21.2 | 2.8 | 5.5 | 3.5 | 40.7 |
| Sy2-14 | 24.2 | 1.8 | 22.9 | 1.5 | 2.6 | 2.7 | 44.3 |
| Sy2-15 | 23.5 | 5.4 | 21.3 | 0.0 | 2.1 | 1.8 | 45.8 |
| Sy2-16 | 26.7 | 5.2 | 16.6 | 0.1 | 0.0 | 3.2 | 48.2 |
| Sy2-17 | 26.9 | 0.9 | 21.0 | 2.0 | 3.5 | 3.4 | 42.4 |
| Sy2-18 | 21.2 | 8.4 | 20.9 | 1.6 | 1.6 | 2.8 | 43.5 |
| Sy2-19 | 29.2 | 2.2 | 18.6 | 2.9 | 6.3 | 2.6 | 38.2 |
| Sy2-20 | 28.5 | 3.6 | 17.7 | 3.9 | 6.0 | 3.6 | 36.8 |
| Sy2-21 | 34.6 | 1.0 | 16.3 | 6.6 | 5.9 | 1.9 | 33.7 |
| Sy2-22 | 27.8 | 0.0 | 23.8 | 4.8 | 5.6 | 4.5 | 33.4 |
| Sy2-23 | 24.3 | 5.5 | 23.9 | 3.8 | 6.7 | 2.9 | 32.8 |
| Sy2-24 | 25.8 | 3.9 | 24.0 | 4.0 | 5.1 | 3.6 | 33.6 |
| Sy2-25 | 24.5 | 8.1 | 18.8 | 1.2 | 5.6 | 4.2 | 37.6 |
| Sy2-26 | 27.8 | 0.0 | 24.9 | 2.6 | 4.4 | 3.8 | 36.6 |
| Sy2-27 | 26.9 | 0.3 | 26.0 | 2.1 | 4.3 | 4.1 | 36.2 |
| Sy2-28 | 30.1 | 1.2 | 25.0 | 1.4 | 5.8 | 1.2 | 35.2 |
| Sy2-29 | 24.5 | 1.3 | 25.2 | 1.8 | 2.5 | 3.9 | 40.8 |
| Sy2-30 | 26.1 | 4.2 | 22.3 | 1.9 | 1.9 | 2.9 | 40.7 |
| Sy2-31 | 29.4 | 6.8 | 15.9 | 0.2 | 1.4 | 4.5 | 41.9 |
| Sy2-32 | 21.3 | 10.9 | 20.7 | 0.6 | 1.0 | 4.3 | 41.3 |
| Sy2-33 | 22.5 | 5.9 | 24.3 | 0.1 | 0.0 | 4.9 | 42.3 |
| Sy2-34 | 26.9 | 5.9 | 21.9 | 1.3 | 1.6 | 2.8 | 39.7 |
| Sy2-35 | 28.7 | 0.8 | 24.4 | 1.5 | 2.3 | 3.7 | 38.7 |
| Sy2-36 | 29.4 | 6.8 | 20.7 | 1.1 | 3.7 | 2.6 | 35.7 |
| Sy2-37 | 30.1 | 1.9 | 24.7 | 0.2 | 0.9 | 3.8 | 38.3 |
| Sy2-38 | 31.2 | 1.1 | 25.1 | 0.2 | 0.1 | 4.6 | 37.7 |
| Sy2-39 | 32.1 | 4.2 | 24.6 | 0.8 | 0.6 | 2.9 | 34.8 |
| Sy2-40 | 33.5 | 6.1 | 21.8 | 1.6 | 0.2 | 3.4 | 33.5 |
| Sy2-41 | 33.4 | 1.6 | 26.8 | 1.8 | 2.9 | 2.7 | 30.8 |
| Sy2-42 | 30.5 | 3.3 | 23.6 | 1.1 | 4.9 | 4.5 | 32.1 |
| Sy2-43 | 25.9 | 2.0 | 28.8 | 7.5 | 0.4 | 2.9 | 32.5 |
| Sy2-44 | 28.4 | 2.3 | 24.5 | 2.9 | 6.1 | 3.5 | 32.3 |
| Sy2-45 | 28.9 | 2.8 | 22.6 | 1.5 | 7.1 | 4.4 | 32.8 |
| Sy2-46 | 24.7 | 5.7 | 23.7 | 3.7 | 9.0 | 3.1 | 30.2 |
| Sy2-47 | 34.2 | 7.1 | 20.7 | 2.3 | 6.7 | 2.4 | 26.6 |
| Sy2-48 | 38.7 | 3.6 | 23.5 | 0.5 | 2.2 | 3.8 | 27.7 |
| Sy2-49 | 39.5 | 1.5 | 25.5 | 0.1 | 1.9 | 4.3 | 27.2 |
| Sy2-50 | 39.4 | 5.0 | 22.8 | 0.4 | 0.0 | 3.5 | 29.0 |
| Sy2-51 | 39.7 | 2.1 | 28.5 | 1.5 | 0.5 | 2.4 | 25.3 |
| Sy2-52 | 39.9 | 5.8 | 25.2 | 1.1 | 1.9 | 2.9 | 23.2 |
| Sy2-53 | 40.4 | 8.1 | 19.9 | 5.8 | 8.4 | 3.4 | 14.0 |
| Sy2-54 | 24.8 | 4.5 | 26.0 | 6.5 | 14.4 | 4.6 | 19.3 |
| Sy2-55 | 29.1 | 8.5 | 18.1 | 6.8 | 16.4 | 4.7 | 16.4 |
| Sy2-56 | 14.6 | 0.1 | 12.6 | 7.3 | 57.0 | 3.5 | 4.8 |
| Sy2-57 | 15.3 | 1.1 | 13.7 | 8.3 | 56.1 | 0.7 | 4.8 |
