# Supplementary material for: Pore types, genesis, and evolution model of lacustrine oil-prone shale: a case study of the Cretaceous Qingshankou Formation, Songliao Basin, NE China
Source: Sci Rep. 2022 Oct 14;12:17210. doi: 10.1038/s41598-022-21154-y (PMC9568562; doi:10.1038/s41598-022-21154-y)
Supplement: Supplementary file 2 — Supplementary Information 2. [file 41598_2022_21154_MOESM2_ESM.docx]

| Table S2. Basic properties of shale samples used in this work | | | | | | | | | | | | | | | |
| --- | --- | --- | --- | --- | --- | --- | --- | --- | --- | --- | --- | --- | --- | --- | --- |
| Depth (m) | *R*_o_ (%) | Depth (m) | A/TOC | Depth (m) | K(%) | Depth (m) | I(%) | Depth (m) | I/S(%) | Depth (m) | Calcite(%) | Depth(m) | Median  pore radius  (μm） | Depth (m) | Porosity （%) |
| 1223.89 | 0.72 | 1226.01 | 0.140 | 1200.26 | 40.39 | 1203.00 | 49.46 | 1203.59 | 2.22 | 1201.20 | 5.81 | 1201.26 | 0.53 | 1297.17 | 21.29 |
| 1234.64 | 0.72 | 1270.20 | 0.192 | 1200.33 | 7.30 | 1203.07 | 43.55 | 1203.59 | 4.72 | 1202.40 | 9.03 | 1201.38 | 0.61 | 1305.57 | 26.70 |
| 1339.76 | 0.74 | 1346.80 | 0.210 | 1200.39 | 41.40 | 1204.60 | 15.59 | 1204.79 | 6.94 | 1202.40 | 12.90 | 1201.44 | 0.90 | 1309.17 | 4.07 |
| 1369.62 | 0.72 | 1356.15 | 0.306 | 1200.47 | 60.96 | 1205.57 | 33.87 | 1204.79 | 8.89 | 1203.59 | 1.62 | 1202.54 | 0.72 | 1312.77 | 8.66 |
| 1386.35 | 0.52 | 1369.79 | 0.114 | 1201.53 | 72.19 | 1212.88 | 22.58 | 1223.93 | 4.99 | 1203.59 | 2.91 | 1293.53 | 0.58 | 1342.76 | 18.00 |
| 1407.85 | 0.87 | 1399.64 | 0.150 | 1201.60 | 66.85 | 1235.01 | 71.49 | 1226.32 | 7.49 | 1203.59 | 24.84 | 1296.07 | 1.30 | 1349.96 | 16.94 |
| 1483.11 | 0.80 | 1411.39 | 0.246 | 1205.43 | 47.59 | 1235.30 | 47.30 | 1229.91 | 3.04 | 1233.53 | 1.31 | 1296.42 | 3.06 | 1351.16 | 9.15 |
| 1567.92 | 0.76 | 1484.63 | 0.167 | 1208.57 | 85.56 | 1236.34 | 60.74 | 1231.11 | 9.71 | 1233.53 | 5.82 | 1300.31 | 4.56 | 1418.34 | 17.33 |
| 1701.71 | 0.50 | 1590.16 | 0.107 | 1234.35 | 36.36 | 1236.43 | 53.21 | 1238.29 | 5.54 | 1238.32 | 13.89 | 1302.30 | 2.54 | 1420.74 | 13.56 |
| 1762.63 | 0.98 | 1707.38 | 0.169 | 1235.62 | 30.48 | 1248.75 | 22.56 | 1360.09 | 1.60 | 1250.30 | 3.89 | 1304.86 | 3.36 | 1424.34 | 10.44 |
| 1792.49 | 0.85 | 1708.31 | 0.282 | 1240.72 | 5.35 | 1360.39 | 84.89 | 1392.65 | 1.04 | 1350.90 | 3.94 | 1306.10 | 3.58 | 1463.92 | 22.89 |
| 1836.69 | 0.88 | 1764.07 | 0.501 | 1241.17 | 67.91 | 1389.52 | 47.78 | 1395.04 | 4.37 | 1355.69 | 0.39 | 1306.68 | 0.49 | 1466.32 | 8.63 |
| 1935.84 | 0.95 | 1780.48 | 0.649 | 1380.74 | 33.69 | 1391.85 | 53.15 | 1450.09 | 2.68 | 1355.69 | 8.78 | 1311.18 | 5.02 | 1467.52 | 14.36 |
| 1991.98 | 0.98 | 1787.83 | 0.082 | 1384.52 | 19.25 | 1396.46 | 67.13 | 1453.68 | 1.01 | 1364.07 | 22.33 | 1314.96 | 0.04 | 1468.72 | 11.25 |
| 1994.37 | 1.12 | 1796.47 | 0.475 | 1391.93 | 1.07 | 1401.43 | 51.54 | 1453.68 | 5.46 | 1378.44 | 12.99 | 1316.29 | 0.62 | 1477.12 | 11.41 |
| 2007.51 | 1.12 | 1846.62 | 0.536 | 1433.59 | 28.34 | 1402.38 | 72.50 | 1474.02 | 2.95 | 1388.02 | 1.05 | 1316.36 | 0.94 | 1478.32 | 13.38 |
| 2029.01 | 1.14 | 1880.93 | 0.214 | 1439.78 | 12.30 | 1441.82 | 73.56 | 1477.61 | 0.17 | 1389.22 | 3.31 | 1318.61 | 0.23 | 1483.12 | 11.90 |
| 2045.73 | 1.12 | 1993.50 | 0.214 | 1476.16 | 80.21 | 1443.45 | 37.00 | 1489.57 | 10.44 | 1395.21 | 6.54 | 1320.31 | 2.77 | 1483.12 | 16.00 |
| 2061.26 | 1.18 | 1999.67 | 0.136 | 1476.28 | 70.05 | 1444.71 | 32.16 | 1490.77 | 16.55 | 1431.14 | 25.27 | 1321.41 | 2.28 | 1484.32 | 14.52 |
| 2077.99 | 1.15 | 2021.06 | 0.206 | 1477.43 | 74.33 | 1480.63 | 27.31 | 1495.56 | 11.55 | 1443.11 | 0.11 | 1348.55 | 0.22 | 1486.72 | 18.62 |
| 2098.29 | 1.22 | 2032.89 | 0.267 | 1485.28 | 19.79 | 1481.21 | 78.39 | 1503.93 | 7.66 | 1494.61 | 11.75 | 1352.21 | 0.58 | 1486.72 | 19.28 |
| 2111.43 | 0.46 | 2038.12 | 0.084 | 1494.82 | 24.60 | 1481.88 | 22.47 | 1505.13 | 2.66 | 1495.81 | 7.23 | 1369.05 | 0.94 | 1486.72 | 21.41 |
| 2180.72 | 0.98 | 2043.51 | 0.337 | 1496.33 | 0.10 | 1483.12 | 18.71 | 1506.32 | 0.12 | 1497.01 | 4.65 | 1454.00 | 0.52 | 1487.92 | 22.23 |
| 2216.55 | 0.54 | 2049.93 | 0.154 | 1503.04 | 39.57 | 1498.01 | 73.54 | 1507.52 | 3.77 | 1498.20 | 16.91 | 1454.04 | 0.74 | 1489.12 | 24.36 |
| 2240.44 | 0.97 | 2061.55 | 0.302 | 1504.58 | 10.70 | 1498.04 | 71.39 | 1549.40 | 0.13 | 1499.40 | 24.98 | 1455.16 | 0.35 | 1490.32 | 2.06 |
| 2264.33 | 1.00 | 2063.17 | 0.127 | 1508.09 | 18.72 | 1501.71 | 64.40 | 1550.60 | 13.19 | 1500.60 | 0.83 | 1483.94 | 0.55 | 1588.69 | 12.86 |
| 2307.34 | 1.43 | 2070.90 | 0.398 | 1547.74 | 13.37 | 1501.82 | 55.26 | 1552.99 | 11.80 | 1552.10 | 5.32 | 1484.03 | 1.00 | 1589.89 | 11.55 |
| 2316.89 | 1.56 | 2080.09 | 0.564 | 1549.02 | 6.95 | 1506.58 | 56.87 | 1554.19 | 3.75 | 1553.29 | 25.00 | 1485.11 | 0.42 | 1591.09 | 14.83 |
| 2343.17 | 1.39 | 2080.88 | 0.232 | 1560.57 | 43.85 | 1509.28 | 31.06 | 1556.58 | 5.97 | 1555.69 | 2.42 | 1495.81 | 0.01 | 1592.29 | 16.79 |
|  |  | 2083.81 | 0.511 | 1563.05 | 37.43 | 1510.41 | 36.44 | 1558.97 | 9.02 | 1561.68 | 0.16 | 1499.54 | 0.68 | 1593.49 | 17.29 |
|  |  | 2089.42 | 0.668 | 1589.67 | 18.18 | 1512.61 | 52.03 | 1593.68 | 17.07 | 1580.84 | 0.50 | 1500.73 | 0.65 | 1594.69 | 18.76 |
|  |  | 2093.41 | 0.503 | 1594.03 | 54.55 | 1512.71 | 43.97 | 1594.87 | 0.40 | 1602.40 | 0.83 | 1502.30 | 2.54 | 1594.69 | 19.91 |
|  |  | 2099.83 | 0.320 | 1594.10 | 48.66 | 1543.36 | 80.51 | 1597.26 | 7.07 | 1656.29 | 0.11 | 1503.36 | 1.82 | 1653.47 | 14.98 |
|  |  | 2104.37 | 0.424 | 1595.57 | 26.74 | 1551.98 | 59.54 | 1598.46 | 14.29 | 1661.08 | 6.34 | 1579.04 | 2.99 | 1804.63 | 4.12 |
|  |  | 2106.27 | 0.634 | 1612.18 | 41.71 | 1553.26 | 52.02 | 1602.05 | 2.62 | 1662.28 | 3.12 | 1584.53 | 0.51 | 1804.63 | 7.07 |
|  |  | 2106.41 | 0.573 | 1612.63 | 4.28 | 1555.36 | 76.75 | 1616.41 | 4.84 | 1662.28 | 20.21 | 1589.43 | 1.07 | 1810.63 | 9.69 |
|  |  | 2107.46 | 0.136 | 1613.71 | 13.90 | 1561.25 | 84.27 | 1618.80 | 2.33 | 1664.67 | 26.99 | 1591.63 | 0.06 | 1814.22 | 5.92 |
|  |  | 2117.76 | 0.337 | 1624.08 | 50.27 | 1593.01 | 27.27 | 1631.97 | 5.11 | 1677.84 | 5.38 | 1597.73 | 0.64 | 1815.42 | 12.32 |
|  |  | 2138.51 | 0.172 | 1632.82 | 21.39 | 1593.71 | 68.67 | 1634.36 | 3.99 | 1680.24 | 4.09 | 1667.15 | 0.41 | 1828.62 | 14.44 |
|  |  | 2151.38 | 0.800 | 1656.97 | 8.56 | 1595.30 | 35.87 | 1642.74 | 3.99 | 1681.44 | 0.10 | 1807.18 | 0.01 | 1831.02 | 16.57 |
|  |  | 2152.80 | 0.207 | 1799.81 | 0.00 | 1599.89 | 52.00 | 1660.68 | 5.65 | 1691.02 | 26.68 | 1812.02 | 0.21 | 1832.22 | 4.93 |
|  |  | 2158.06 | 0.512 | 1810.00 | 50.20 | 1606.91 | 64.90 | 1745.64 | 2.01 | 1692.22 | 22.16 | 1814.59 | 1.09 | 1844.22 | 1.49 |
|  |  | 2158.79 | 0.207 | 1812.00 | 6.42 | 1609.37 | 58.98 | 1793.50 | 3.38 | 1795.21 | 12.53 | 1816.91 | 0.70 | 1844.22 | 17.72 |
|  |  | 2159.82 | 0.276 | 1814.00 | 2.00 | 1621.60 | 35.86 | 1800.68 | 0.87 | 1797.60 | 5.44 | 1822.93 | 0.90 | 1879.01 | 10.51 |
|  |  | 2174.50 | 0.146 | 1830.00 | 9.00 | 1624.89 | 61.13 | 1807.86 | 3.09 | 1803.59 | 1.57 | 1823.96 | 0.02 | 1880.21 | 11.81 |
|  |  | 2200.79 | 0.172 | 1870.00 | 8.00 | 1625.24 | 31.56 | 1809.06 | 6.98 | 1820.36 | 14.16 | 1824.05 | 0.47 | 1881.41 | 12.94 |
|  |  | 2213.71 | 0.277 | 1900.59 | 0.54 | 1629.93 | 39.62 | 1815.04 | 13.09 | 1821.56 | 0.61 | 1828.83 | 0.44 | 1989.37 | 4.41 |
|  |  | 2220.01 | 0.146 | 1932.36 | 52.41 | 1639.30 | 55.75 | 1836.58 | 10.86 | 1827.54 | 1.90 | 1843.34 | 1.13 | 1991.77 | 2.93 |
|  |  | 2237.60 | 0.303 | 1933.84 | 28.88 | 1648.97 | 46.60 | 1846.15 | 9.19 | 1827.54 | 19.64 | 1848.06 | 0.80 | 1991.77 | 6.86 |
|  |  | 2246.34 | 0.155 | 1937.60 | 16.04 | 1718.02 | 71.31 | 1848.55 | 14.74 | 1840.72 | 23.84 | 1860.33 | 2.23 | 1992.97 | 1.78 |
|  |  | 2298.02 | 0.077 | 1946.83 | 45.99 | 1722.68 | 81.52 | 1856.92 | 0.57 | 1840.72 | 26.75 | 1863.64 | 0.83 | 2003.77 | 3.75 |
|  |  | 2302.64 | 0.146 | 1950.53 | 37.97 | 1810.56 | 87.47 | 1862.91 | 3.63 | 1895.81 | 4.84 | 1870.65 | 0.05 | 2109.34 | 2.25 |
|  |  | 2309.62 | 0.234 | 1953.20 | 15.51 | 1814.02 | 42.55 | 1894.02 | 8.89 | 1958.08 | 0.03 | 1971.39 | 0.67 | 2110.54 | 0.05 |
|  |  | 2316.62 | 0.312 | 1953.96 | 51.87 | 1814.67 | 65.54 | 1898.80 | 5.83 | 1958.08 | 2.61 | 1984.54 | 0.54 | 2111.74 | 8.48 |
|  |  | 2317.04 | 0.138 | 1955.08 | 58.29 | 1820.79 | 59.66 | 1916.75 | 5.55 | 1961.68 | 5.51 | 1989.32 | 0.47 | 2114.14 | 9.95 |
|  |  | 2322.75 | 0.251 | 1956.61 | 31.02 | 1828.62 | 82.65 | 1926.32 | 0.20 | 1976.05 | 0.36 | 1996.48 | 0.34 | 2121.34 | 11.92 |
|  |  | 2322.90 | 0.190 | 1964.59 | 65.78 | 1830.24 | 65.00 | 1937.09 | 14.43 | 1979.64 | 14.55 | 2007.19 | 0.01 | 2122.54 | 6.01 |
|  |  | 2338.49 | 0.182 | 1966.10 | 40.11 | 1835.15 | 59.65 | 1943.08 | 3.87 | 1984.43 | 18.10 | 2010.83 | 0.27 | 2122.54 | 7.16 |
|  |  | 2354.52 | 0.487 | 1984.48 | 8.02 | 1849.54 | 58.58 | 1947.86 | 0.81 | 1985.63 | 3.59 | 2022.87 | 0.57 | 2122.54 | 13.39 |
|  |  | 2357.50 | 0.243 | 1996.42 | 12.83 | 1850.85 | 53.76 | 1952.65 | 7.76 | 1991.62 | 27.78 | 2032.55 | 1.06 | 2123.74 | 16.34 |
|  |  | 2359.01 | 0.112 | 2083.39 | 0.10 | 1852.16 | 48.95 | 1962.22 | 4.14 | 1998.80 | 5.85 | 2053.90 | 0.05 | 2124.94 | 2.73 |
|  |  | 2366.87 | 0.330 | 2109.47 | 23.94 | 1860.28 | 59.64 | 1968.21 | 1.36 | 2009.58 | 26.50 | 2054.12 | 1.15 | 2124.94 | 4.37 |
|  |  | 2367.00 | 0.278 | 2112.02 | 11.70 | 1870.40 | 86.91 | 1982.56 | 2.19 | 2013.17 | 6.83 | 2065.94 | 0.34 | 2129.73 | 8.64 |
|  |  |  |  | 2113.29 | 5.32 | 1879.27 | 66.05 | 1983.76 | 5.25 | 2040.72 | 2.00 | 2066.01 | 0.73 | 2141.73 | 19.12 |
|  |  |  |  | 2128.67 | 22.34 | 1891.01 | 75.67 | 1987.35 | 29.69 | 2034.31 | 43.94 | 2067.08 | 0.08 | 2142.93 | 16.17 |
|  |  |  |  | 2128.73 | 17.55 | 1902.56 | 43.05 | 1993.33 | 15.52 | 2069.46 | 6.85 | 2067.48 | 2.07 | 2169.32 | 6.66 |
|  |  |  |  | 2130.95 | 32.45 | 1904.81 | 48.93 | 1995.73 | 28.02 | 2070.66 | 10.08 | 2068.79 | 2.62 | 2183.72 | 7.80 |
|  |  |  |  | 2132.44 | 7.45 | 1910.21 | 73.53 | 1998.12 | 8.02 | 2071.86 | 0.72 | 2070.37 | 4.54 | 2190.92 | 11.90 |
|  |  |  |  | 2133.57 | 13.83 | 1910.39 | 66.04 | 2007.69 | 1.90 | 2071.86 | 15.24 | 2070.94 | 1.41 | 2223.31 | 2.06 |
|  |  |  |  | 2134.91 | 1.60 | 1925.28 | 43.57 | 2086.67 | 3.26 | 2079.04 | 21.05 | 2079.28 | 1.19 | 2240.10 | 4.51 |
|  |  |  |  | 2199.64 | 1.06 | 1927.00 | 71.92 | 2086.67 | 14.65 | 2080.24 | 14.28 | 2081.80 | 1.84 | 2254.50 | 4.02 |
|  |  |  |  | 2205.57 | 6.92 | 1929.27 | 76.73 | 2098.63 | 4.64 | 2085.03 | 6.54 | 2086.25 | 0.11 | 2280.89 | 2.86 |
|  |  |  |  | 2209.20 | 3.72 | 1941.59 | 62.28 | 2099.83 | 15.48 | 2087.43 | 27.83 | 2091.10 | 0.40 | 2283.29 | 6.14 |
|  |  |  |  | 2246.30 | 8.51 | 1942.50 | 74.05 | 2102.22 | 7.70 | 2088.62 | 0.09 | 2100.63 | 0.14 | 2283.29 | 8.60 |
|  |  |  |  | 2247.42 | 15.43 | 1943.58 | 78.86 | 2109.40 | 9.36 | 2093.41 | 10.09 | 2124.57 | 0.11 | 2292.89 | 7.29 |
|  |  |  |  | 2313.55 | 0.00 | 1946.87 | 41.43 | 2114.19 | 2.97 | 2097.01 | 2.99 | 2128.21 | 0.34 | 2326.48 | 12.69 |
|  |  |  |  | 2499.10 | 18.09 | 1947.81 | 52.12 | 2117.78 | 5.75 | 2098.20 | 46.87 | 2129.47 | 0.66 | 2330.08 | 17.77 |
|  |  |  |  | 2246.30 | 8.51 | 1952.94 | 87.95 | 2120.17 | 0.47 | 2110.18 | 24.94 | 2129.49 | 0.76 | 2342.07 | 11.70 |
|  |  |  |  |  |  | 1955.09 | 47.84 | 2124.96 | 2.13 | 2111.38 | 1.07 | 2183.33 | 0.50 | 2342.07 | 18.59 |
|  |  |  |  |  |  | 1961.35 | 36.61 | 2124.96 | 7.97 | 2112.57 | 7.52 | 2190.42 | 0.01 | 2347.67 | 17.27 |
|  |  |  |  |  |  | 1961.69 | 72.44 | 2126.15 | 11.30 | 2112.57 | 27.52 | 2198.91 | 0.53 | 2351.67 | 13.17 |
|  |  |  |  |  |  | 1961.83 | 66.55 | 2133.33 | 9.63 | 2113.77 | 37.20 | 2202.44 | 0.20 | 2354.07 | 6.94 |
|  |  |  |  |  |  | 1965.70 | 54.79 | 2134.53 | 5.19 | 2117.37 | 9.78 | 2220.42 | 0.30 | 2355.27 | 8.42 |
|  |  |  |  |  |  | 1966.13 | 86.87 | 2147.69 | 43.79 | 2122.16 | 13.01 | 2234.83 | 0.50 | 2368.47 | 9.73 |
|  |  |  |  |  |  | 1995.96 | 90.07 | 2168.03 | 10.17 | 2122.16 | 31.07 | 2251.60 | 0.50 | 2429.65 | 1.68 |
|  |  |  |  |  |  | 1998.73 | 74.56 | 2168.03 | 14.34 | 2125.75 | 38.49 | 2258.71 | 0.14 | 2458.44 | 7.74 |
|  |  |  |  |  |  | 2000.88 | 84.72 | 2184.79 | 6.00 | 2128.14 | 3.01 | 2100.63 | 0.14 | 2283.29 | 8.60 |
|  |  |  |  |  |  | 2001.39 | 63.33 | 2188.38 | 12.94 | 2131.74 | 10.75 | 2124.57 | 0.11 | 2292.89 | 7.29 |
|  |  |  |  |  |  | 2007.80 | 45.68 | 2189.57 | 3.77 | 2132.93 | 29.14 | 2128.21 | 0.34 | 2326.48 | 12.69 |
|  |  |  |  |  |  | 2007.93 | 90.07 | 2195.56 | 35.72 | 2132.93 | 44.30 | 2129.47 | 0.66 | 2330.08 | 17.77 |
|  |  |  |  |  |  | 2009.93 | 56.38 | 2199.15 | 20.72 | 2134.13 | 0.22 | 2129.49 | 0.76 | 2342.07 | 11.70 |
|  |  |  |  |  |  | 2015.63 | 68.14 | 2200.34 | 0.10 | 2136.53 | 6.56 | 2183.33 | 0.50 | 2342.07 | 18.59 |
|  |  |  |  |  |  | 2017.64 | 84.18 | 2201.54 | 6.27 | 2143.71 | 45.79 | 2190.42 | 0.01 | 2347.67 | 17.27 |
|  |  |  |  |  |  | 2101.96 | 61.15 | 2202.74 | 3.49 | 2162.87 | 29.15 | 2198.91 | 0.53 | 2351.67 | 13.17 |
|  |  |  |  |  |  | 2114.29 | 95.91 | 2207.52 | 11.82 | 2168.86 | 3.67 | 2202.44 | 0.20 | 2354.07 | 6.94 |
|  |  |  |  |  |  | 2120.45 | 88.42 | 2208.72 | 13.77 | 2173.65 | 0.45 | 2220.42 | 0.30 | 2355.27 | 8.42 |
|  |  |  |  |  |  | 2122.97 | 83.07 | 2225.47 | 2.37 | 2178.44 | 13.68 | 2234.83 | 0.50 | 2368.47 | 9.73 |
|  |  |  |  |  |  | 2131.29 | 85.21 | 2230.26 | 20.70 | 2182.04 | 28.84 | 2251.60 | 0.50 | 2429.65 | 1.68 |
|  |  |  |  |  |  | 2131.51 | 76.12 | 2235.04 | 5.98 | 2183.23 | 12.39 | 2267.18 | 0.59 |  |  |
|  |  |  |  |  |  | 2131.58 | 73.44 | 2239.83 | 0.14 | 2185.63 | 50.00 | 2276.81 | 0.82 |  |  |
|  |  |  |  |  |  | 2133.03 | 62.75 | 2263.76 | 20.69 | 2189.22 | 9.49 | 2336.71 | 0.92 |  |  |
|  |  |  |  |  |  | 2134.56 | 48.84 | 2266.15 | 1.80 | 2194.01 | 28.52 | 2336.91 | 1.93 |  |  |
|  |  |  |  |  |  | 2137.37 | 81.46 | 2267.35 | 6.24 | 2197.60 | 6.91 | 2337.84 | 0.59 |  |  |
|  |  |  |  |  |  | 2137.96 | 56.86 | 2269.74 | 16.24 | 2200.00 | 13.36 | 2339.20 | 1.38 |  |  |
|  |  |  |  |  |  | 2138.38 | 88.95 | 2270.94 | 11.80 | 2202.40 | 3.69 | 2339.24 | 1.57 |  |  |
|  |  |  |  |  |  | 2167.41 | 76.10 | 2278.12 | 28.18 | 2204.79 | 0.18 | 2341.35 | 0.17 |  |  |
|  |  |  |  |  |  | 2174.70 | 71.29 | 2285.30 | 17.90 | 2211.98 | 16.60 | 2351.29 | 1.96 |  |  |
|  |  |  |  |  |  | 2180.83 | 65.40 | 2286.50 | 7.35 | 2211.98 | 34.02 | 2352.32 | 1.11 |  |  |
|  |  |  |  |  |  | 2181.68 | 79.84 | 2288.89 | 0.68 | 2217.96 | 35.31 | 2353.28 | 0.06 |  |  |
|  |  |  |  |  |  | 2181.85 | 72.89 | 2298.46 | 6.23 | 2221.56 | 27.89 | 2353.40 | 0.53 |  |  |
|  |  |  |  |  |  | 2184.53 | 60.59 | 2298.46 | 10.12 | 2222.75 | 14.34 | 2354.56 | 0.36 |  |  |
|  |  |  |  |  |  | 2185.55 | 68.07 | 2303.25 | 16.51 | 2223.95 | 12.09 | 2361.85 | 0.85 |  |  |
|  |  |  |  |  |  | 2186.16 | 92.67 | 2316.41 | 3.45 | 2229.94 | 8.86 | 2366.77 | 1.51 |  |  |
|  |  |  |  |  |  | 2186.29 | 87.32 | 2316.41 | 7.34 | 2231.14 | 6.93 | 2367.88 | 1.08 |  |  |
|  |  |  |  |  |  | 2186.34 | 85.19 | 2317.61 | 12.06 | 2232.34 | 0.80 | 2491.11 | 0.46 |  |  |
|  |  |  |  |  |  | 2196.19 | 73.42 | 2317.61 | 24.83 | 2232.34 | 25.31 |  |  |  |  |
|  |  |  |  |  |  | 2197.43 | 71.81 | 2324.79 | 13.72 | 2238.32 | 23.38 |  |  |  |  |
|  |  |  |  |  |  | 2234.85 | 57.89 | 2346.32 | 10.38 | 2249.10 | 0.48 |  |  |  |  |
|  |  |  |  |  |  | 2235.63 | 75.54 | 2378.63 | 8.98 | 2249.10 | 13.06 |  |  |  |  |
|  |  |  |  |  |  | 2237.91 | 80.35 | 2379.83 | 5.37 | 2251.50 | 18.87 |  |  |  |  |
|  |  |  |  |  |  | 2265.45 | 79.27 | 2428.89 | 2.29 | 2252.69 | 9.52 |  |  |  |  |
|  |  |  |  |  |  | 2277.33 | 83.01 | 2430.09 | 9.23 | 2253.89 | 5.00 |  |  |  |  |
|  |  |  |  |  |  | 2278.64 | 78.20 | 2487.52 | 8.38 | 2256.29 | 3.07 |  |  |  |  |
|  |  |  |  |  |  | 2279.09 | 59.48 | 2492.31 | 5.04 | 2264.67 | 13.39 |  |  |  |  |
|  |  |  |  |  |  | 2285.49 | 92.10 | 2536.58 | 4.75 | 2275.00 | 50.00 |  |  |  |  |
|  |  |  |  |  |  | 2289.96 | 55.20 | 2555.73 | 8.91 | 2265.87 | 44.04 |  |  |  |  |
|  |  |  |  |  |  | 2299.15 | 71.24 | 2556.92 | 5.02 | 2268.26 | 5.33 |  |  |  |  |
|  |  |  |  |  |  | 2300.27 | 74.45 |  |  | 2280.24 | 32.11 |  |  |  |  |
|  |  |  |  |  |  | 2305.47 | 57.33 |  |  | 2285.03 | 29.21 |  |  |  |  |
|  |  |  |  |  |  | 2310.60 | 92.62 |  |  | 2286.23 | 24.37 |  |  |  |  |
|  |  |  |  |  |  | 2313.65 | 65.35 |  |  | 2294.61 | 0.83 |  |  |  |  |
|  |  |  |  |  |  | 2314.49 | 80.32 |  |  | 2300.60 | 26.96 |  |  |  |  |
|  |  |  |  |  |  | 2319.42 | 74.44 |  |  | 2301.80 | 30.19 |  |  |  |  |
|  |  |  |  |  |  | 2320.98 | 58.93 |  |  | 2308.98 | 14.71 |  |  |  |  |
|  |  |  |  |  |  | 2326.13 | 93.69 |  |  | 2311.38 | 12.13 |  |  |  |  |
|  |  |  |  |  |  | 2330.13 | 76.57 |  |  | 2328.14 | 29.88 |  |  |  |  |
|  |  |  |  |  |  | 2332.40 | 81.92 |  |  | 2332.93 | 0.85 |  |  |  |  |
|  |  |  |  |  |  | 2338.60 | 72.83 |  |  | 2334.13 | 5.04 |  |  |  |  |
|  |  |  |  |  |  | 2342.33 | 66.94 |  |  | 2342.51 | 0.12 |  |  |  |  |
|  |  |  |  |  |  | 2345.61 | 79.78 |  |  | 2346.11 | 8.59 |  |  |  |  |
|  |  |  |  |  |  | 2356.62 | 69.61 |  |  | 2347.31 | 3.76 |  |  |  |  |
|  |  |  |  |  |  | 2370.36 | 95.81 |  |  | 2353.29 | 0.11 |  |  |  |  |
|  |  |  |  |  |  | 2390.26 | 64.25 |  |  | 2362.87 | 3.44 |  |  |  |  |
|  |  |  |  |  |  | 2395.97 | 75.48 |  |  | 2364.07 | 23.76 |  |  |  |  |
|  |  |  |  |  |  | 2427.96 | 88.84 |  |  | 2403.59 | 17.97 |  |  |  |  |
|  |  |  |  |  |  | 2430.21 | 94.72 |  |  | 2403.59 | 37.33 |  |  |  |  |
|  |  |  |  |  |  | 2497.62 | 78.11 |  |  | 2417.96 | 2.17 |  |  |  |  |
|  |  |  |  |  |  | 2501.44 | 68.49 |  |  | 2445.51 | 4.77 |  |  |  |  |
|  |  |  |  |  |  | 2540.36 | 92.00 |  |  | 2482.63 | 7.04 |  |  |  |  |
|  |  |  |  |  |  | 2547.73 | 83.98 |  |  | 2499.40 | 5.12 |  |  |  |  |
|  |  |  |  |  |  | 2561.90 | 91.99 |  |  |  |  |  |  |  |  |
